# Case report: Heart failure secondary to myocardial infarction in a fertile woman with woven coronary artery

**DOI:** 10.3389/fcvm.2022.1034860

**Published:** 2022-11-07

**Authors:** Jiayi Hu, Qian Wang, Mei Dong, Huixia Lu

**Affiliations:** ^1^The Key Laboratory of Cardiovascular Remodeling and Function Research, The State and Shandong Province Joint Key Laboratory of Translational Cardiovascular Medicine, Department of Cardiology, Qilu Hospital of Shandong University, Chinese Ministry of Education, Chinese National Health Commission and Chinese Academy of Medical Sciences, Jinan, China; ^2^The Key Laboratory of Cardiovascular Remodeling and Function Research, The State and Shandong Province Joint Key Laboratory of Translational Cardiovascular Medicine, Department of Radiology, Qilu Hospital of Shandong University, Chinese Ministry of Education, Chinese National Health Commission and Chinese Academy of Medical Sciences, Jinan, China

**Keywords:** heart failure, ischemic heart disease, woven coronary artery, coronary angiography, coronary artery bypass grafting

## Abstract

A 42-year-old female was hospitalized with 2-day history of fever, dyspnea, and chest tightness. Four years ago, she had similar symptoms at the eighth week of gestation. Computed tomography coronary angiography only suggested moderate stenosis, but cardiac MRI indicated myocardial infarction. The coronary angiography demonstrated a woven coronary artery. She underwent successful coronary artery bypass grafting surgery and took medication regularly. Finally, the prognosis was favorable. Cardiovascular events seldom happen in fertile women because of the protection of estrogen, but once it occurs, the potential cause, such as coronary anomaly and other risk factors, should not be overlooked.

## Introduction

Woven coronary artery (WCA) is a rare congenital vascular variation with unknown etiology, in which the epicardial coronary artery is divided into channels that bifurcate and weave along the axis of the coronary artery and then merge again as the main coronary lumen (1, 2). Although previous cases were usually detected incidentally and WCA was considered a benign anomaly because of its normal blood flow (3), the challenge for physicians is to recognize, which cases in fact may attribute to the possibility of angina (4), acute coronary syndrome (5, 6) even sudden cardiac death (7). However, to date, WCA associated with heart failure (HF) secondary to myocardial infarction (MI) has been not reported, especially in fertile women who have a lower incidence of cardiovascular events.

## Case description

A 42-year-old woman visited the clinic with a 2-day history of fever (39.3^°^C), complaining of recurrent dyspnea, chest tightness, reduced exercise capacity and dizziness.

The symptoms, which could be relieved by rest, first afflicted her 4 years prior at 8 weeks of gestation and continued, even after her full-term normal delivery. Since then, she had noticed frequently and progressively worsening dyspnea and chest tightness over the last 10 months. She had primary thrombocytosis, grade 2 hypertension (160–179/100–109 mmHg) with very high absolute total cardiovascular risk (CVR) and adenomyosis. However, she denied diabetes, coronary artery disease (CAD), relevant family history or smoking history. Her record showed that only the primary thrombocytosis had been treated regularly, with hydroxyurea. Electrocardiography (ECG) demonstrated poor R wave progression in the anterior wall. Transthoracic echocardiography (TTE) indicated impaired left ventricular (LV) contractility, with a reduced left ventricular ejection fraction (LVEF) of 32% and an enlarged left ventricular end-diastole diameter (LVEDD) of 67 mm (Table 1). Segmental LV hypokinesis was also observed in her TTE. Furthermore, computed tomography coronary angiography (CTCA) revealed diffuse wall thickening in the left main (LM) and left anterior descending (LAD) coronary arteries and moderate stenosis in the proximal segment of the LAD (Supplementary Figure 1). Additionally, laboratory tests showed that N-terminal pro-brain natriuretic peptide (NT-pro BNP) was raised to 1,118 pg/ml, low-density lipoprotein cholesterol (LDL-C) to 2.16 mmol/L and the platelet (PLT) count to 535 × 10^9^/L, while hemoglobin (Hb) was lowered to 87 g/L (Table 2).

**TABLE 1:** Transthoracic echocardiography (TTE) results.

Parameters	Results
Left atria anteroposterior diameter (LAD)	38 mm
Left ventricular end-diastole diameter (LVEDD)	67 mm
Right atria longitudinal diameter (RAL) × transverse diameter (RAT)	44 mm × 51 mm
Right ventricle end-diastole diameter (RVEDD)	27 mm
Interventricular septum thickness (IVS)	10 mm
Left ventricular posterior wall (LVPW)	9 mm
Left ventricular ejection fraction (LVEF)	0.32
Pulmonary arterial systolic pressure (PASP)	38 mmHg
The ratio of early diastolic mitral in flow velocity (E) to early diastolic TDI annular velocity’s (E/e’)	14

**TABLE 2:** Lab tests results.

Lab test	Results	Reference value
Hemoglobin (HGB)	87 g/L	130–175 g/L
Platelets (PLT)	535 × 10^9^/L	115–150 × 10^9^/L
Cardiac troponin I (cTnI)	10.05 ng/L	
N-terminal B-type natriuretic peptide (NT-proBNP)	1,118 pg/mL	≤125 pg/mL
Alanine aminotransferase (ALT)	25 U/L	90–50 U/L
Aspartate aminotransferase (AST)	30 U/L	15–40 U/L
Creatinine (Cr)	104 μmol/L	62–115 μmol/L
Uric acid (UA)	409 μmol/L	208–420 μmol/L
Homocysteine (Hcy)	11 μmol/L	<15 μmol/L
Total cholesterol (T-cho)	3.66 mmol/L	2.80–6.00
Low-density lipoprotein cholesterol (LDL-C)	2.16 mmol/L	1.00–3.37
Fasting blood-glucose (FBG)	4.68 mmol/L	3.90–6.10
C-reactive protein (CRP)	<3.02 mg/L	<8.00 mg/L

Given all these findings, a preliminary diagnosis of HF (NYHA class II) and CAD was made. Coronary angiography (CAG) was repeatedly recommended, but the patient requested conservative treatment because of concerns about the risks associated with invasive detection. She was given anti-HF drugs (betaloc 190 mg qd, spironolactone 20 mg qd, and sacubitril/valsartan 200 mg bid), antiplatelet agents (aspirin 100 mg qd and hydroxyurea 500 mg qd) and cholesterol-reducing medicine (atorvastatin 20 mg qn). However, after such guideline-directed medical therapy (GDMT), the patient’s symptoms could not be fully alleviated for more than half a year.

**FIGURE 1:**
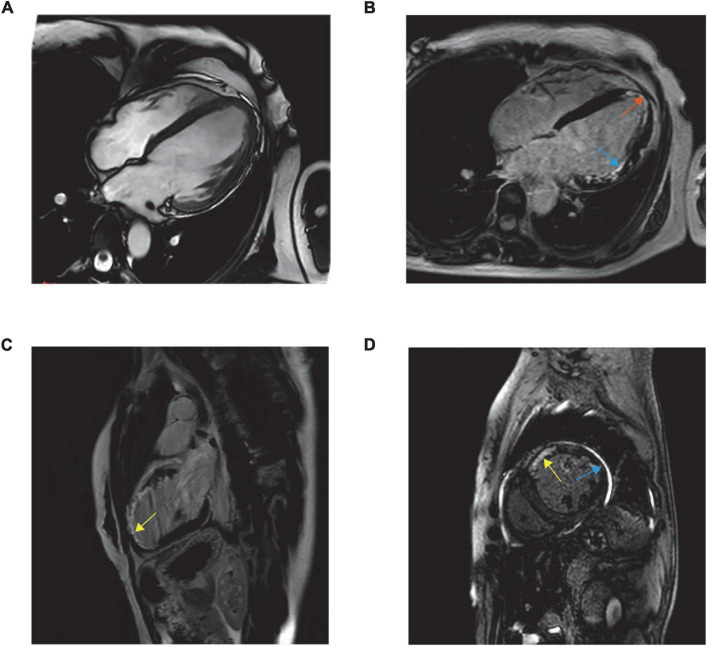
Balanced steady-state free precession MR images are obtained in standard cardiac planes. Long-axis and four-chamber view **(A)**, long-axis and four-chamber late gadolinium-enhanced MR image **(B)**, long-axis and two-chamber view **(C)**, and short-axis and two-chamber view **(D)**.

The poor clinical response promoted further imaging (Figure 1). Cardiac magnetic resonance imaging (MRI) indicated LV dilation (66 mm), thinning of the left ventricle region wall (3 mm) and abnormal motion and suggested transmural infarction at the apex (orange arrow) and the lateral wall (blue arrow) and partial infarction at the anterior wall (yellow arrow).

According to the following factors, HF secondary to MI became the most likely diagnosis: risk factors for atherosclerosis and hypertension existed, although they are more common in elderly people; thrombocytosis increased the risk of platelet aggregation and thrombus formation, and the level of PLT was still not controlled, which led to the high incidence of thrombosis. ECG reflected myocardial necrosis or injury. Segmental LV hypokinesis was a characteristic sign of myocardial ischemia and MI in TTE. CTA only showed moderate stenosis, but the cardiac MRI indicated MI.

**FIGURE 2:**
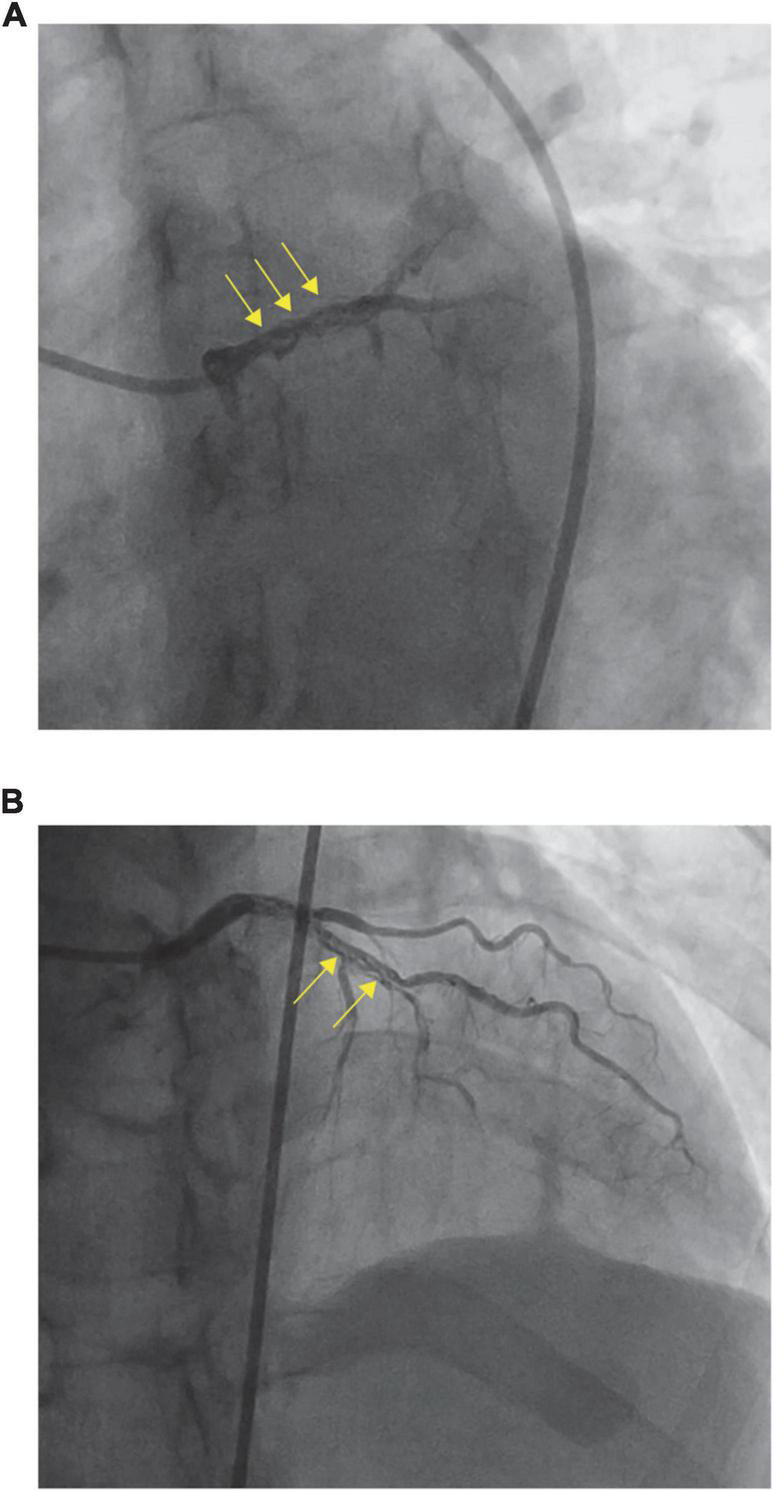
Coronary angiography (CAG) **(A,B)** revealed woven coronary artery (WCA).

To identify the possible etiological factors, the patient underwent urgent CAG for further investigation, which revealed that the LM and proximal LAD looked woven—they split into multiple slender arteries that intertwined and twisted along the axis of the coronary artery—and were fully occluded except for the D1 branch (Figure 2). CAG visualized the vascular morphology, and WCA was preliminarily diagnosed. As CAG showed distal occlusion, intravascular imaging, such as intravenous ultrasound (IVUS) and optical coherence tomography (OCT), was not selected because of the possibility of vascular injury caused by interventional operation.

Coronary artery bypass grafting (CABG) may be safer than stent implantation based on intravascular imaging because the presence of stenosis or thrombosis is predicted to be associated with a higher risk of complications from percutaneous coronary intervention (PCI) (5, 8, 9). In the present case, because the laboratory indicators were unsatisfactory, monitoring of the patient’s signs and parameters was also essential. After the surgery, she adhered to regular medications. With the continuation of the previous treatment, her cardiac function improved significantly.

To date, the patient has not complained of obvious discomfort. Her cardiac systolic function improved significantly, and her LVEF recovered to 53% on TTE 6 months after CABG.

## Discussion

The pathogenesis of WCA has not been elucidated, but most scholars believe that a congenital malformation is the most likely cause. As we have increasingly learned, WCA is not a completely benign disease: it may be asymptomatic, or it may be accompanied by chest pain, myocardial ischemia, myocardial infarction and even sudden cardiac death. Although women of childbearing age tend to have fewer cardiovascular events, it is essential to confirm the diagnosis and to exclude the differential diagnosis by further examinations in a timely manner once symptoms and signs are found. In this case, the patient presented with obvious symptoms of HF, which were consistent with relevant laboratory tests. Therefore, we needed to identify diseases that have similar symptoms, signs and test results.

Myocarditis often results from viral infection, presents with a wide range of symptoms, ranging from mild dyspnea or chest pain that resolves without specific therapy to cardiogenic shock and sudden death, and even has dilated cardiomyopathy with chronic heart failure as the long-term sequela. Since the patient had fever, we suspected myocarditis. However, the virus tests were negative, and no significant signs of viral infection were detected 4 years prior.

Peripartum cardiomyopathy (PPCM) is common in the child-bearing period and should be suspected in any peripartum women presenting with symptoms or signs of HF and with confirmed left ventricular dysfunction. However, in most cases, it occurs toward the end of pregnancy or in the months following delivery, rather than the first trimester of pregnancy or a few years after good pregnant outcomes (10).

Dilated cardiomyopathy (DCM) represents a particular etiology of systolic HF that frequently has a genetic background and usually affects young patients with few comorbidities (11). DCM usually occurs with a genetic background and affects young patients; however, this patient did not have a family history, and genetic screening suggested that no pathogenic gene was found. Additionally, DCM does not show segmental dyskinesia of the ventricular wall on echocardiography or MRI.

Anemic heart disease needs to be considered because the patient had a history of adenomyosis with pain and heavy menstrual bleeding, and her Hb had dropped to 87 g/L. Patients with long-term chronic anemia whose Hb is close to or lower than 70 g/L should be suspected of having anemic heart disease. Fortunately, our patient did not meet this precondition. Under the circumstances of not correcting Hb by using IV iron, erythropoietin or other treatments, the patient returned to our hospital for routine blood tests 6 months later, and we noted a significant increase in her Hb (101 g/L). Therefore, this diagnosis could also be set aside.

Some theories believe that WCA may be a consequence of spontaneous coronary artery dissection (SCAD) or recanalized thrombus. And the patient did feel sick during peripartum. We considered that women of childbearing age were less likely to have atherosclerotic heart disease and no atherosclerosis was in other blood vessels, so we still thought that WCA was the cause of MI rather than the effect. And CAG indicated that the channel of woven arteries had single lumen with three layers. But recanalized thrombus divided the lumen into multiple small cavities communicating with each other, while the true arterial lumen of SCAD compressed by the false lumen at several points, and both of them had no separate three-layered structure could be defined. In fact, it is difficult to make a differential diagnosis by CAG alone, but because IVUS and OCT are not suitable for the patient, we can only try our best to make a reasonable diagnosis by CAG.

The vast majority of cases of WCA are detected by CAG, by which filling/defects and reticular or braided structures can be seen in the lumen of the coronary artery involved. Using acoustic imaging features, IVUS can display complete cross-sectional images of blood vessels and evaluate the blood vessel diameter and lumen cross-sectional area more accurately, which is more conducive to the diagnosis of WCA. With the development of endovascular imaging technology, OCT can clearly observe the internal structure of the coronary artery due to its higher resolution. It has become the “gold standard” for the diagnosis of WCA and is used for the definitive diagnosis of preliminary suspicious cases after CAG. However, IVUS and OCT are not suitable for severe vascular tortuosity or stenosis.

The scope and clinical manifestations of WCA vary from person to person, so there are no clear guidelines for treatment at present. In general, if the patients have no symptoms of MI and the coronary flow reserve and distal vascular function are normal, only follow-up observation is needed (12). However, if symptoms are present, imaging examinations, fraction flow reserve (FFR) and instantaneous wave-free ratio (iFR) should be performed to accurately evaluate the distal blood flow function to make a reasonable judgment on the choice of medications or PCI or CABG (5, 8). If stenosis or thrombosis is seen, PCI may have a higher risk of complications, so CABG may be safer than stent implantation based on intravascular imaging (9).

## Data availability statement

The original contributions presented in this study are included in the article/Supplementary material, further inquiries can be directed to the corresponding author.

## Ethics statement

Written informed consent was obtained from the individual(s) for the publication of any potentially identifiable images or data included in this article.

## Author contributions

JH and MD participated in the study design and drafted the manuscript. MD contributed to the data collection. JH was responsible for writing the manuscript. QW, MD, and HL contributed to the manuscript revision. All authors contributed to the article and approved the submitted version.
