# Supplementary figures and images for: Case report: Heart failure secondary to myocardial infarction in a fertile woman with woven coronary artery
Source: Front Cardiovasc Med. 2022 Nov 7;9:1034860. doi: 10.3389/fcvm.2022.1034860 (PMC9676242; doi:10.3389/fcvm.2022.1034860)

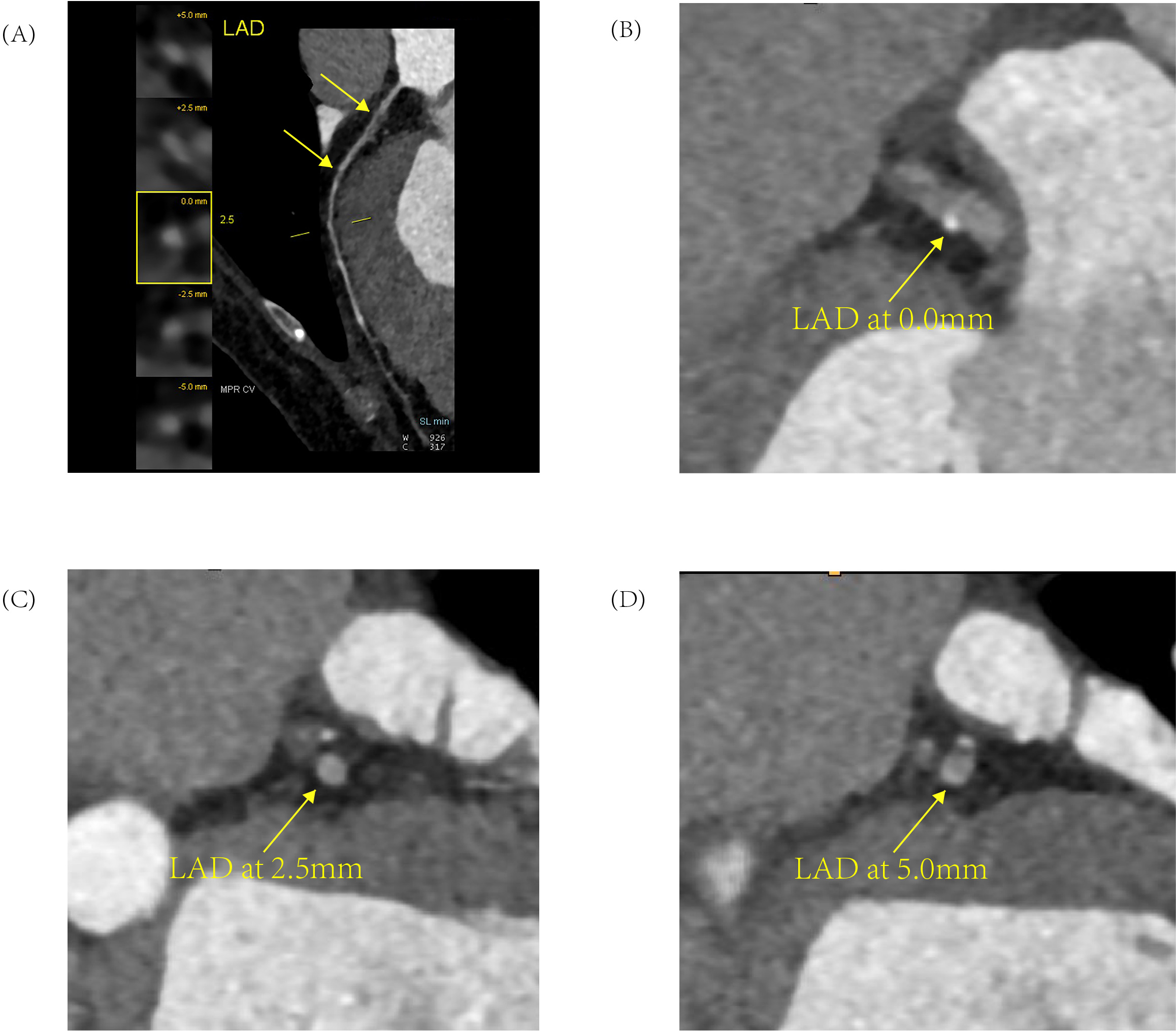

Supplement: Supplementary file 1 [file Image_1.JPEG]

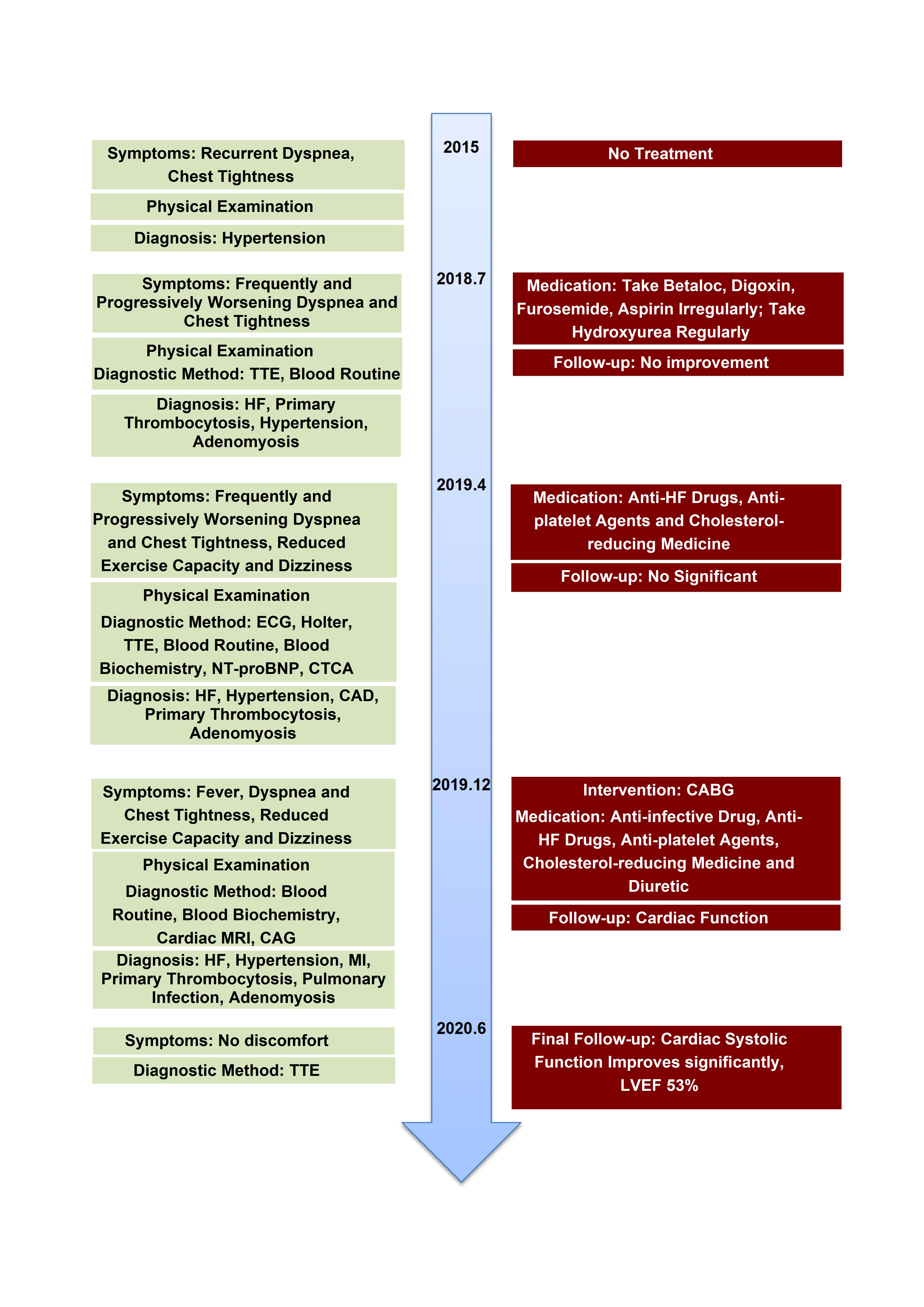

Supplement: Supplementary file 2 [file Image_2.JPEG]
